# Web 2.0 Tools in the Prevention of Curable Sexually Transmitted Diseases: Scoping Review

**DOI:** 10.2196/jmir.8871

**Published:** 2018-03-22

**Authors:** María Sanz-Lorente, Carmina Wanden-Berghe, Ramón Castejón-Bolea, Javier Sanz-Valero

**Affiliations:** ^1^ Department of Public Health & History of Science School of Medicine University Miguel Hernandez of Elche Alicante Spain; ^2^ Foundation for the Promotion of Health and Biomedical Research from the Valencian Community University General Hospital of Alicante Alicante Spain

**Keywords:** sexually transmitted diseases, bacterial, internet, webcasts, social media

## Abstract

**Background:**

The internet is now the primary source of information that young people use to get information on issues related to sex, contraception, and sexually transmitted infections.

**Objective:**

The goal of the research was to review the scientific literature related to the use of Web 2.0 tools as opposed to other strategies in the prevention of curable sexually transmitted diseases (STDs).

**Methods:**

A scoping review was performed on the documentation indexed in the bibliographic databases MEDLINE, Cochrane Library, Scopus, Cumulative Index to Nursing and Allied Health Literature, Web of Science, Literatura Latinoamericana y del Caribe en Ciencias de la Salud, PsycINFO, Educational Resources Information Center, the databases of Centro Superior de Investigaciones Científicas in Spain, and the Índice Bibliográfico Español de Ciencias de la Salud from the first available date according to the characteristics of each database until April 2017. The equation search was realized by means of the using of descriptors together with the consultation of the fields of title register and summary with free terms. Bibliographies of the selected papers were searched for additional articles.

**Results:**

A total of 627 references were retrieved, of which 6 papers were selected after applying the inclusion and exclusion criteria. The STDs studied were chlamydia, gonorrhea, and syphilis. The Web 2.0 tools used were Facebook, Twitter, Instagram, and YouTube. The 6 papers used Web 2.0 in the promotion of STD detection.

**Conclusions:**

Web 2.0 tools have demonstrated a positive effect on the promotion of prevention strategies for STDs and can help attract and link youth to campaigns related to sexual health. These tools can be combined with other interventions. In any case, Web 2.0 and especially Facebook have all the potential to become essential instruments for public health.

## Introduction

General measures of health promotion and education are fundamental in the prevention of sexually transmitted diseases (STDs), especially favorable strategies for safe sex. Health education on the symptoms of these diseases, methods of transmission, prevention, diagnosis, and treatment are main measures of control [1].

STDs have profound effects on sexual and reproductive health worldwide and are among the 5 major categories for which adults seek health care. Every day more than 1 million people contract a sexually transmitted infection. It is estimated that annually, about 357 million people contract any of 4 curable STDs: chlamydia (131 million), gonorrhea (78 million), syphilis (5.6 million), or trichomoniasis (143 million) [2].

In the 21st century, the epidemiological evolution of STDs cannot be understood without taking into account factors such as globalization, migration, and the information and communication technologies (ICTs) that have led to new approaches in the study of their transmission and prevention [3]. As a result, sex education must be addressed from all facets of possible action making sure that the information is complete. In this last aspect, neither school nor the family seems to be sufficient [4]. The usual methods used in education for sexuality, such as workshops and presentations, among others, play an important role in the transfer of information; however, today's young adult has different interests. In this regard, Jimenez [5] states that “young people have a constant provision to the use and management, contact and utilization of technological gadgets; taking these to make them partakers of their life in whatever place and moment required.”

As for health education, it is necessary that the content system and messages related to the prevention reach young people in the most informal and entertaining way, for which ICTs would be very useful. It is well known that the dawn of Web 2.0 resources has provoked a substantive change in the communication of knowledge, favoring its disclosure by enabling the expansion and permeability of knowledge at a very low cost. Web 2.0 has shown its integration in today’s information society and, far from dwindling, increasingly has more initiatives that enhance it, subsequently contributing to the diffusion of the contents about health [6].

In Spain, in a survey conducted by Doctoralia internet in 2016 [7], young people between 18 and 24 years old were the most prone to self-medication (41%), and 7% of them have made a mistake by choosing a medication or searching for a solution for their health problem on the internet. At the time, 69% of this group sought information on the internet after being diagnosed with a condition. One-quarter (26%) confessed to having lied or hidden information from their doctor, doing so because they didn't want to reveal some aspect of their intimacy, they felt they had done something wrong to their health, or they felt shame at the time of appointment or consultation, especially with the urologist (21%).

Despite easy access to health professionals in specialized units, many young girls get their information from friends and on the Web; the internet is now the primary source of information that young people use to get information on issues related to sex, contraception, and sexually transmitted infections [8]. The vast majority of teenagers search on the internet because of its anonymity without taking into account that not everything they find will be true [9].

Information can improve people's ability to recognize the symptoms of STDs, increasing the chances that they will request medical attention or encourage their partners to do so [2].

In this context, the objective of this systematic review was to evaluate studies that use the Web 2.0 in contrast with other strategies to prevent curable STDs.

## Methods

Data were obtained from the following bibliographic databases in the field of health science: MEDLINE (via PubMed), Cochrane Library, Scopus, Cumulative Index to Nursing and Allied Health Literature, Web of Science, *Literatura Latinoamericana y del Caribe en Ciencias de la Salud*, PsycINFO, Educational Resources Information Center, the bibliographic databases of the *Centro Superior de Investigaciones Científicas* of Spain, and the *Índice Bibliográfico Español de Ciencias de la Salud*.

### Information Processing

Search terms were chosen from the thesaurus developed by the US National Library of Medicine (Medical Subject Headings [MeSH] and title/abstract), and the final search syntax was shaped by the Boolean intersection of 2 equations (equation 1 AND equation 2):

- Equation 1: (“internet”[MeSH] OR “Social Media”[MeSH] OR “internet”[Title/Abstract] OR “World Wide Web”[Title/Abstract] OR “WWW”[Title/Abstract] OR “Web”[Title/Abstract] OR “Social Media”[Title/Abstract] OR “Blog”[Title/Abstract] OR “Wikipedia”[Title/Abstract] OR “Wiki”[Title/Abstract] OR “YouTube”[Title/Abstract] OR “Facebook”[Title/Abstract] OR “Twitter”[Title/Abstract])
- Equation 2: (“Sexually Transmitted Diseases, Bacterial”[MeSH] OR “Trichomonas Infections”[MeSH] OR “Bacterial Sexually Transmitted Disease”[Title/Abstract] OR “Sexually Transmitted Diseases, Bacterial”[Title/Abstract] OR “Bacterial STIs”[Title/Abstract] OR “Bacterial STDs”[Title/Abstract] OR “Bacterial Venereal Disease”[Title/Abstract] OR “Bacterial Sexually Transmitted Infection”[Title/Abstract] OR “Venereal Diseases, Bacterial”[Title/Abstract] OR “Chancroid”[Title/Abstract] OR “Lymphogranuloma Venereum”[Title/Abstract] OR “Trachoma”[Title/Abstract] OR “Chlamydia”[Title/Abstract] OR “Chlamydia Infection”[Title/Abstract] OR “Gonorrhea”[Title/Abstract] OR “Neisseria”[Title/Abstract] OR “Granuloma Inguinale”[Title/Abstract] OR “Granuloma Venereum”[Title/Abstract] OR “*Haemophilus ducreyi* ”[Title/Abstract] OR “Donovanosis”[Title/Abstract] OR “Syphilis”[Title/Abstract] OR “Treponema”[Title/Abstract] OR “Great Pox”[Title/Abstract] OR “Chancre”[Title/Abstract] OR “*Klebsiella granulomatis*”[Title/Abstract] OR “Calymmatobacterium”[Title/Abstract] OR “*Mycoplasma genitalium*”[Title/Abstract] OR “*Ureaplasma urealyticum*”[Title/Abstract] OR “*Trichomonas vaginalis*”[Title/Abstract] OR “Trichomonas Infection”[Title/Abstract] OR “Trichomonas vaginitis”[Title/Abstract])

The final search equation was developed for use in the database MEDLINE, via PubMed, using the filters: “Humans” and “Comparative Study” or “Evaluation Studies.”

This strategy was adapted to the characteristics of each of the rest of the databases consulted. The search was carried out from the first available date according to the characteristics of each database until April 2017 and was completed with the consideration of the bibliographic listing of the items that were selected.

### Final Selection of Papers

Papers were selected that met the following criteria (criteria of inclusion): comply with the objectives of the search, published in journals reviewed by peers, and written in English, Spanish, Portuguese, French, or German. Papers that did not present results about the advantages of Web 2.0 in relation to other strategies for the prevention of curable STDs were excluded.

The selection of the relevant papers was performed independently by 2 authors (MSL and JSV). For inclusion of the studies, it was established that the valuation of the concordance between these authors (kappa index) must be greater than .80. Provided this condition is fulfilled, possible discrepancies were solved through consultation with the author CWB and subsequent consensus among all the authors [10].

The quality of the selected documents was evaluated using the Strengthening the Reporting of Observational Studies in Epidemiology (STROBE) guidelines [11], which contain a list of 22 essential items that must be described in the studies. For each selected paper, 1 point was assigned for each present item (not applicable=0). When an item addressed several issues, these were evaluated independently, giving partial value to each one and averaging so that in no case could the value be more than one.

### Data Extraction

Control of the correctness of the data was performed using double tables that allowed the detection of deviations and their correction by revising the originals. The semiperiod of Burton-Kebler (the median of age) and Price (percentage of papers less than 5 years old) indices were calculated to determine the relevance of papers. The studies were grouped according to the variables to study in order to systematize and facilitate the understanding of the results, considering the following data: first author of the bibliographic reference and year of publication, type of study, country and age of the participants, curable STD discussed, Web 2.0 tool used in the study, period in which the work was done, intervention carried out, and results obtained.

## Results

A total of 627 references were retrieved, and 1 paper was obtained from the bibliographic listings of relevant retrieved papers.

**Figure 1:**
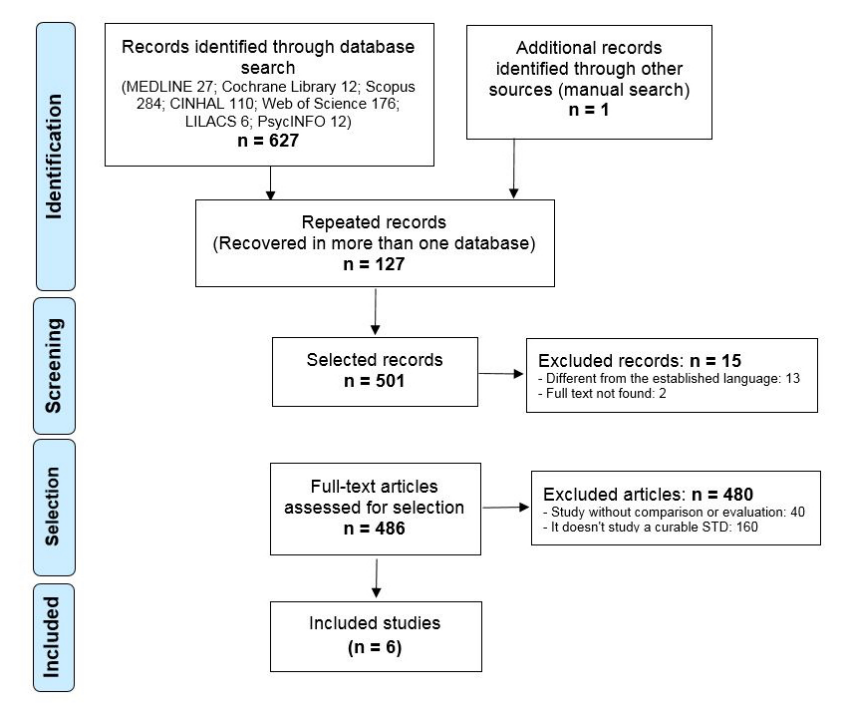
Identification and selection of studies.

After debugging the duplicates, applying inclusion and exclusion criteria, and consulting the bibliographic lists (see Figure 1), 6 documents [12-17] were selected for review and critical analysis (Multimedia Appendix 1). The calculation of kappa coefficient gave a measure of agreement on the selection of the papers, between evaluators, of .96 (*P*<.001).

The 6 selected papers presented an obsolescence, according to the Burton-Kebler index, equal to 1 year, with a Price index of 100%. When assessing the quality of papers selected for review using the STROBE questionnaire, scores ranged between 8.33 and 17.00, with a median of 13.51 (Multimedia Appendix 2).

The revised works were 3 evaluation studies [12,15,16] and 3 comparative studies [13,14,17]; 5 developed in the United States [12,14-17] and 1 in New Zealand [13]; all written in English.

All studies were developed in people aged 25 years or less except Habet et al [14], which included participants up to 35 years old in its second phase. The curable STD targets of these works were chlamydia [12,14,16,17], gonorrhea [12,14,17], syphilis [12,13], and any STD [15].

The longest period of implementation of a promotion about STD testing was the Get Yourself Tested (GYT) campaign [18] through the Division of Sexually Transmitted Disease Prevention, Centers for Disease Control and Prevention (CDC), observed in Friedman et al [15].

Facebook was the Web 2.0 tool used in the 6 papers, although Dowshen et al [12] also used Twitter, Instagram, and YouTube and Friedman et al [15] also used Twitter. The results of user interactions were offered in 3 works: Dowshen et al [12] noted approximately 6000 visits to Facebook and 128 likes, 46 followers on Twitter, 390 YouTube views, and 42 Instagram followers; interaction data in Friedman et al [15] offered 4477 Facebook likes and 1994 Twitter followers; and Bull et al [17] indicated on average 43 visits per week (range of 37 to 101).

The Dowshen et al [12], Coughlan et al [13], and Friedman et al [15] papers determined the usefulness of Web 2.0 tools for increasing awareness in and implementation of screening. Jones et al [16] and Bull et al [17] identified an increase in condom use and positive changes in behavior among the participant population as a result of the promotion campaign. On the contrary, Habel et al [14] did not observe favorable differences in relation to testing, indicating that it would have been a key to the training and collaboration of health care personnel in support of the campaign. The Dowshen et al [12] and Bull et al [17] papers also reported a reduction in positive cases.

## Discussion

### Principal Findings

The results of this review show that Web 2.0 tools can be useful in the prevention of curable STDs. The identification of just 6 publications is not surprising since an exploratory review on the promotion of sexual health through social networks found 51 papers, mostly focusing on HIV infection; no assessment or comparison with traditional methods was performed. In addition, the authors of this work pointed out that most of the efforts to implement ICT in the promotion of STD prevention campaigns had developed in HIV campaigns despite an increase in the incidence of curable STDs [19].

On the other hand, the high number of nonrelevant papers was mainly due to results obtained from the Web of Science and Scopus databases, which do not have descriptor thesauri. Queries are constructed by entering text in title, abstract, and keyword fields. This high documentary noise has been observed in other systematic reviews [20,21].

In the papers selected for review, validity and topicality were verified. The data obtained indicate a lower obsolescence than has been observed in works previously published in the field of health sciences. Moreover, it is evident that the results derived from the age of publication (measured by the median and Price index) is a characteristic of an area of knowledge in full emergence [22].

The fact that the documents included in the review were written in English and came mostly from US institutions was an expected fact in line with the existing bibliometric results [23].

The age of the population included in the reviewed studies coincides with the age group with greater incidence of STDs [24]. Statistics of major international health agencies show that young people are most affected by STDs, and these consequences can affect the rest of their lives. The vast majority declare being sexually active and protect themselves from pregnancy but not from STDs. In general, they show little knowledge of sexual transmission of infections, although they know of the concept. Syphilis is considered a disease of other people. Some knew about gonorrhea but most had not heard of chlamydia and did not perceive themselves to be at risk [25].

Young people say loneliness and abandonment are to blame for the lack of information about their sexuality. Thus, the most frequent source of information is friends, then the internet, traditional media (especially television), parents, and finally medical consultation. University organizations are rarely mentioned, except for sporadic or very specific initiatives [26].

At the same time, of the curable STDs studied, chlamydia, gonorrhea, and syphilis have the highest incidence and prevalence [27], which is an an adequate representation of these diseases.

The presence of Facebook in all the studies reviewed is logical; this Web 2.0 tool has been placed among the 3 most commonly used in the world and has already shown its potential for health promotion [28,29]. As the CDC indicates, Facebook is a tool of great potential for its use in different prevention programs and health promotions [30].

In recent years, Facebook, YouTube, Twitter, and other Web 2.0 tools have become effective ways to expand the reach, promote commitment, and increase access to messages on health and prevention and treatment of STDs [30-32].

Little attention was given to user interaction. Only half of the works described these data (visits, likes, followers, etc) and none assessed them. These data could have provided interesting results complementary to those that could had been obtained through traditional systems of public health surveillance, as is seen in the recent work of Gittleman et al [33]. The essence of the user interaction with the materials of the Web 2.0 lies in knowing the interest generated together with a wide range of services enabling collaboration and fast exchange of information among users of a community or social network.

Users can communicate with the issuers of material and show appreciation through a symbolic, easily understandable code (I like/dislike) or even by emotional expressions (I love it, I enjoy it, it saddens me, etc) in an agile exchange of information facilitated by the structure and design of the website. Research shows that even low user interaction or passivity is something attractive in the content consumer, probably by the distrust generated by not knowing who is on the other side of the screen or, simply, to avoid being observed (monitored) when they have to register (give personal data) to be able to interact with the Web tool [34].

The revised works focused their intervention on the awareness and prevention of STDs, with particular emphasis on the promotion of screening. However, until now there has not been much evidence about how the information on the Web influences people’s health behavior, which is necessary to deepen the study of the usefulness of social networks for the benefit of the promotion of health [35]. Thus, Taggart et al [36] and Hochberg et al [37], in 2 separate systematic reviews on HIV published in 2015, pointed to the need for further research to determine to what extent ICTs can influence the prevention of STDs. This recommendation also appeared in all revised papers [12-17].

### Limitations

A limitation of this review could be the low number of selected papers because it is an area of emerging technological application. It has been stated that systematic reviews should be based on studies with design and selection that ensure greater scientific rigor, but in this analysis, all retrieved papers focusing on the studied subject were included.

According to the US Agency for Health Research and Quality, epidemiological designs of the studies selected in this review do not guarantee full validity and reliability of the obtained observations. However, the evidence available is probably the best, given the difficulties of study in this area of research and based on the observations obtained in the different interventions. As a result, while it would have been more interesting to have a specific questionnaire, it was considered appropriate to use the STROBE questionnaire to evaluate the quality of the studies.

Although the real limitations are due to the characteristic of each study *per se*, from these limitations, important lessons in formulating appropriate actions for the development, implementation, and evaluation of future Web 2.0 applications can be extracted.

### Conclusions

For all of these reasons, we conclude that Web 2.0 tools have demonstrated a positive effect on the promotion of prevention strategies for STDs and can help attract and link young people to campaigns related to sexual health. These tools can even be combined with other interventions. In any case, Web 2.0 tools, especially Facebook, have all the potential to become key instruments in public health.

## Appendix

Multimedia Appendix 1 Characteristics and main findings of the studies selected for review.

Multimedia Appendix 2 Methodological quality of the studies based on the 22-point assessment from the Strengthening the Reporting of Observational Studies in Epidemiology guideline.

## Abbreviations

CDC: Centers for Disease Control and Prevention
ICT: information and communication technology
MeSH: Medical Subject Headings
STD: sexually transmitted disease
STROBE: Strengthening the Reporting of Observational Studies in Epidemiology
