# Supplementary material for: Web 2.0 Tools in the Prevention of Curable Sexually Transmitted Diseases: Scoping Review
Source: J Med Internet Res. 2018 Mar 22;20(3):e113. doi: 10.2196/jmir.8871 (PMC5887040; doi:10.2196/jmir.8871)
Supplement: Multimedia Appendix 1 [file jmir_v20i3e113_app1.pdf]

Multimedia Appendix 1. Characteristics and main findings of the studies selected for review on Web 2.0 tools in the prevention of curable sexually transmitted diseases (CSTDs).

Author, Year	Study Type	Country	Age	Curable STD	2.0 Tools	Period	Intervention made	Obtained results
**Dowshen et al. 2015 [12]**	Evaluation of study	USA	One year before the campaign: 17.23 years<br><br>In campaign: average age 17.53 years	Chlamydia<br>Gonorrhoea<br>Syphilis	Facebook<br>Twitter<br>Instagram<br>YouTube	Previous the campaign 09/2011 – 08/2012<br><br>During campaign 09/2012 – 08/2013	The campaign was announced in social networks and visits to the clinic were compared a year before the campaign and during the campaign.	More than 1,500 individual interactions were due directly to the campaign.<br>CSTD testing was increased substantially in the 2nd period (48.8 vs. 55.6 %, p<0.05).
**Counghlan et al. 2015 [13]**	Comparative case series	New Zealand	>18 years	Syphilis	Facebook	Since 11/2012 – 05/2013	Facebook page for syphilis awareness and where to get tested.	The number of tests was multiplied by 3 from the campaign.<br>No new cases were reported in the 3 months following the campaign.
**Habel et al. 2015 [14]**	Prospective Comparative	USA	Phase 1: >18 years<br><br>Phase 2: 18 to 35 years	Chlamydia<br>Gonorrhoea	Facebook	Phase 1: 02/2011 – 07/2012<br><br>Phase 2: 09/2011 – 08/2012	It was announced on Facebook the place where to obtain the test and was compared with the population that goes to the pharmacy to buy it	Both systems had low recruitment.
**Friedman et al. 2014 [15]**	Evaluation Study	USA	15 to 25 years	CSTD in general	Facebook<br>Twitter	04/2008 – 01/2010	Campaign for the promotion of CSTD tests.	The 71% increase in tests making on CSTD
**Jones et al. 2012 [16]**	Evaluation Study	USA	15 to 24 years	Chlamydia	Facebook	09/2010 – 2011	Information on Facebook about infection due to Chlamydia.	23% increase in the use of the condom.<br>54% reduction in positive cases of chlamydia among young people.
**Bull et al. 2012 [17]**	Comparative	USA	16 to 25 years	Chlamydia<br>Gonorrhoea	Facebook	10/2010 – 05/2011	Information via messages through Facebook	Increase in the use of condoms in the control group (68% *vs* 56%, p=0.04) in CSTD prevention.
