# Supplementary material for: Web 2.0 Tools in the Prevention of Curable Sexually Transmitted Diseases: Scoping Review
Source: J Med Internet Res. 2018 Mar 22;20(3):e113. doi: 10.2196/jmir.8871 (PMC5887040; doi:10.2196/jmir.8871)
Supplement: Multimedia Appendix 2 [file jmir_v20i3e113_app2.pdf]

Multimedia Appendix 2. Methodological quality of the studies through the 22-point assessment of the STROBE Guide.

Article	Score of the questionnaire Items [a]																						
	**1**	**2**	**3**	**4**	**5**	**6**	**7**	**8**	**9**	**10**	**11**	**12**	**13**	**14**	**15**	**16**	**17**	**18**	**19**	**20**	**21**	**22**	**TOTAL**	**%**
Dowshen et al. [12]	0.5	1	1	1	1	1	0	0	0	1	1	0.6	0	0.2	0	0.5	1	1	1	0	1	1	13.80	62.73
Counghlan et al. [13]	0.5	1	1	1	1	1	0	0	0	1	0	0.4	0.66	0.66	0	0	0	1	1	1	1	1	13.22	60.09
Habel et al. [14]	0.5	1	1	1	1	1	0	0	0	1	0	0.2	0.66	0.5	0	0	0	1	0	1	0	0	9.86	44.82
Friedman et al. [15]	0.5	1	0	0	1	0	0	1	0	0	1	0	0.33	0.5	0	0	0	1	1	1	0	0	8.33	37.86
Jones et al. [16]	0.5	1	0	1	1	1	1	1	1	1	1	0.6	0	1	1	1	0	1	1	1	0	0	16.10	73.18
Bull et al. [17]	0.5	1	1	0	1	1	1	1	0	1	1	1	1	0.5	1	1	0	1	1	0	1	1	17.00	77.27

[a] 0 = It doesn't accomplish; 0 to 1 = partially meets the item; 1 = meets the item entirely
